# A rare case of idiopathic tumoral calcinosis: Case report

**DOI:** 10.1016/j.radcr.2022.08.038

**Published:** 2022-09-15

**Authors:** Khadija Laasri, Salma El houss, Ismail Mohamed Halfi, Ittimade Nassar, Nabil Moatassim Billah

**Affiliations:** Department of Radiology, Ibn Sina University Hospital, Mohamed V University, Rabat, Morocco

**Keywords:** Tumor calcinosis, Idiopathic, Hip, Periarticular calcifications, MRI

## Abstract

Idiopathic tumoral calcinosis is a very uncommon benign disease, defined by the presence of calcified deposits in periarticular tissues. The pathogenesis is yet not well understood. The treatment remains essentially surgical and the prognosis is very good. We report a case of tumoral calcinosis in a 50-year-old patient presenting with a firm mass of soft tissues and limitation of motion of his right hip joint. The diagnosis was first hand suggested radiologically due to the presence of voluminous periarticular calcifications with no bone involvement, and later on confirmed in pathology results. Therefore, imaging continues to be the best modality in order to assess the extension, evaluate the prognosis, and select the adequate surgical approach.

## Introduction

Idiopathic tumoral calcinosis is a unique hereditary metabolic dysfunction of phosphate regulation associated with development of massive periarticular calcinosis in the extra-capsular soft tissues. Typically occurring around large joints, commonly hip, elbow, and shoulder, and can be associated with palpable mass near the affected joint. In this report, we ought to underline the role of radiology in diagnosing this type of entities with an accompanied review of literature.

## Case report

We report the case of a 50-year-old white male, was admitted with a chief complaint of limitation of motion of his right hip joint, with a palpable mass in the same region. The patient stated he had been aware of a mass in his right hip for 4 years which caused only minimal dull pain after strenuous exertion, but had a limitation of motion in his right lower extremity for 1 year. Otherwise, the patient had been in good health, and he denied any history of trauma. Physical examination revealed a firm, hard mass about the right hip. There was moderate limitation of motion of the right hip, with some atrophy and weakness of the right lower extremity musculature, and the remainder of his physical examination was within normal limits. Initial laboratory analysis was normal.

**Fig. 1:**
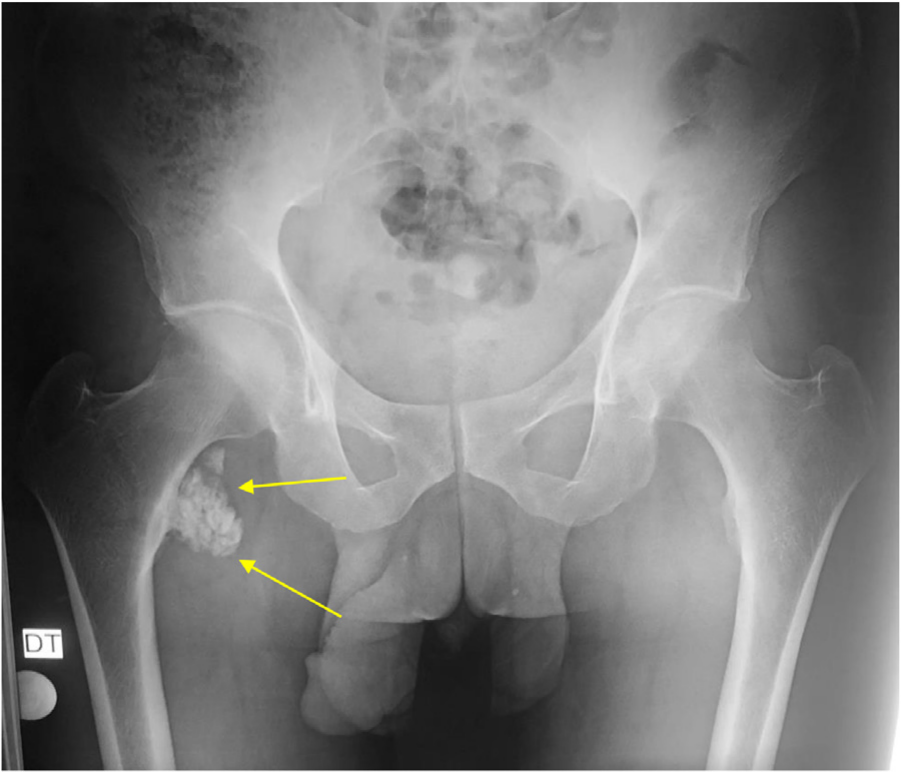
The plain radiographs of the pelvis reveals multiple high-density opacities consistent with soft-tissue calcifications opposite the left greater trochanter without osseous lesion (yellow arrows).

**Fig. 2:**
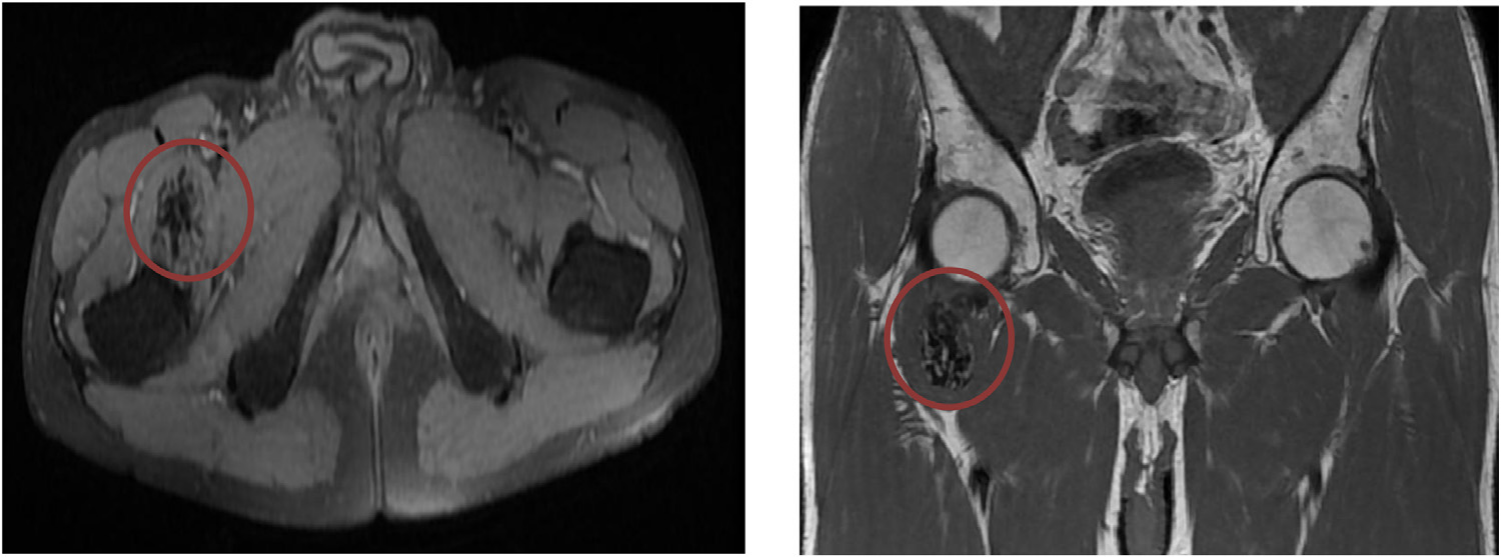
MRI of the pelvis on T1-weighted axial and coronal MRI, showing a lobulated mass of the right hip, well-delimited, and infiltrating the muscles, in hyposignal T1 (red circle).

**Fig. 3:**
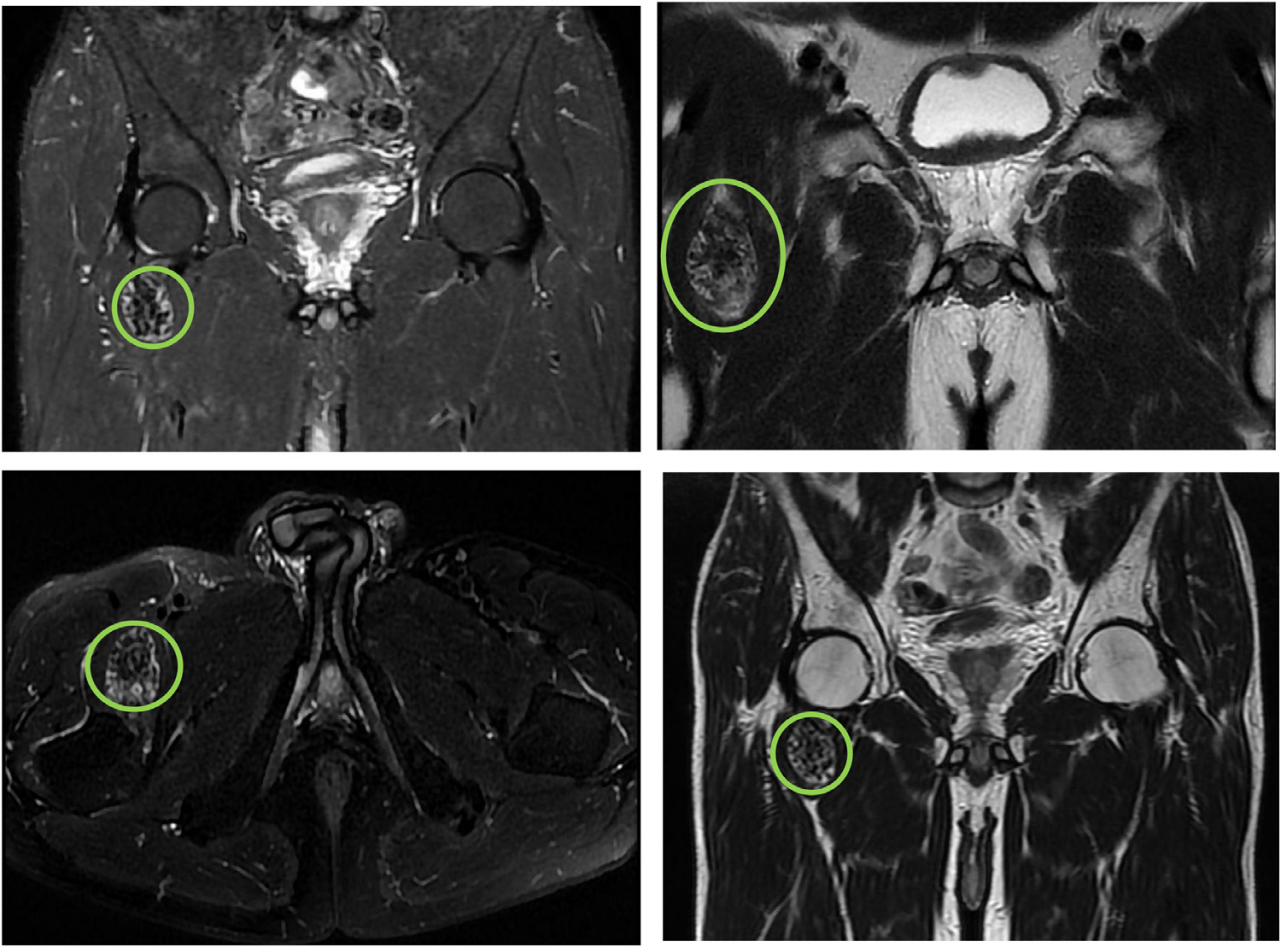
MRI of the pelvis on T2-weighted and fat sat axial and coronal image, the lesion shows a heterogeneous hypersignal with central areas of hyposignal (green circle).

The radiographic of the pelvis showed multiple high-density opacities consistent with soft tissue calcifications were detected on plain radiographs of the right hip (Fig. 1). MRI demonstrated the extension of the soft-tissue; there were no erosions of the femur and iliac bone. The mass lesion was hypointense on T1W images, while it was heterogeneously hyperintense with cystic portions on T2W images (Fig. 2). Multiple fluid-fluid levels in cystic parts of the mass lesion were detected on T2W and fat-saturated T2W images. The inferior part of the level which was hypointense represented the calcium component (Fig. 3). The diagnostic of tumoral calcinosis was suggested by imaging and was confirmed by the anatomopathological exam. Surgery has been successfully done and the some of the mobility of the lamb has been recovered with prescribed follow-up physiotherapy.

## Discussion

The term “tumoral calcinosis” was used historically for the first time by Inclan in 1943 to describe this tumor-like aspect of the calcium deposits in such cases. So far around 200 isolated reports have been published about similar cases [1].

In term of its epidemiology, the idiopathic form usually occurs in the first 3 decades of life with no apparent sex predominance and an apparent predilection towards black populations mostly [2].

Three forms had been identified: (1) an autosomal recessive mutation in one of 3 genes (fibroblast growth factor (FGF) 23, KL, and GALTN3) which induces errors in phosphate metabolism, a complication of renal failure and dialysis, and (3) a sporadic occurrence [3].

On clinical evaluation, it presents as a slow growing soft tissue mass, painless in most cases, arising near large joints, with the hip joint being the most common site in the reported series. The other sites are the elbow, shoulder, knee, wrist, hand and foot [2]. Laboratory analysis most likely return with no significant abnormalities as witnessed in our patient.

On X-rays, it features consist on a well-defined calcified mass located in the periarticular soft tissue, commonly on the extensor side of the articulation. The multilobulated aspect is actually the conglomeration of multiple round or oval opacities with different size and density. The lobules are separated by radiolucent lines, which correlate histologically to the fibrous septa [4].

On MRI, tumoral calcinosis is seen as a well-circumscribed multicystic mass. On T1-weighted images, the mass appears inhomogeneous and has intermediate to low signal intensity (Fig. 2). Interestingly, the lesion displays heterogeneous and relatively high signal intensity on T2-weighted images, despite the large calcium component (Fig. 3). A low signal intensity of the entire lesion in all sequences has been described [2,4]. The septa separating the cysts have low signal on T1-weighted images, variable signal on T2-weighted images and enhance after Gadolinium injection. The inner layers of the septa can hold calcified incrustations, which explain the low signal on both T1- and T2-weighted images. The outer layers are composed of connective tissue associated to a variable degree of vascularization and inflammatory reaction, accounting for the high intensity present on T2-weighted and on postcontrast T1-weighted images [4]. All these features were concluded in our case.

Computed tomography is most of the time not indicated in such cases. The lesion may consist mainly of large cystic components with low attenuation centers and thin layers of calcium outlining the walls. More commonly, there is a more nodular calcified component separated by low-attenuation septations. Some of the septa may enhance after contrast injection. CT clearly demonstrates that the lesion is separated from the bone and shows associated osseous anomalies when present [4], [5], [6].

The ultrasound can show lesion, which is not very calcified. Typically, the lesion appears as a multiloculated mass with multiple cavities limited by echoic thin septa. Some of these septa may be vascularized as shown by the Doppler. Cavities are filled with anechoic or echoic fluid. When the lesions are entirely calcified, they appear as a hyperechogenic mass with an acoustic shadow [2,[4], [5], [6].

The main mimikers of soft tissue calcification, such as heterotopic ossification (myositis ossificans), calcified hemangioma or lymphangioma, teratoma, parosteal osteosarcoma, chronic renal failure, hypervitaminosis D, and the list goes on. Such diseases are excluded by history and laboratory findings in our patient, with the pathology study having the final say in the diagnosis confirmation [2].

The treatment remains symptomatic and palliative mostly. Surgical removal is a common option although few cases described a spontaneous. On the other hand, an inadequate excision leads to a high level of recurrence in patients with and without metabolic disturbances, and growth of recurrent masses is frequently more rapid than that of the initial lesions [5].

## Conclusion

Tumoral calcinosis is a rare disease that can be primary or secondary, and it has a distinct radiographic appearance on conventional radiography, CT, and MRI. The treatment remains essentially surgical and the prognosis is usually positive.

## Authors’ contributions

All authors contributed to this work. All authors have read and approved the final version of the manuscript.

## Patient consent

Written informed consent for publication was obtained from patient.
